# The destiny of an ace: Algimantas Otanas Narakas (1927–1993)

**DOI:** 10.1186/1749-7221-8-6

**Published:** 2013-07-01

**Authors:** Chihab Taleb, Eric Nectoux, Therese Awada, Phillippe Liverneaux

**Affiliations:** 1 Hand Surgery Department, Strasbourg University Hospitals, Illkirch 67403, France; 2 Département d’Orthopédie et Chirurgie de l’Enfant, Pôle enfant, CHRU Lille, 59037,Lille Cedex, France

## 

A complete history of brachial plexus surgery could not be written without the inclusion of Algimantas Narakas. Not only did he define the fundamentals, he also imagined its future development. This year, 2013, marks the 20^th^ anniversary of his death. However, the Narakas Club, which bears his name, continues to keep his heritage very much alive.

Algimantas Otanas Narakas was born on March 23^rd^, 1927 in Kaunas to an aristocratic Lithuanian family. His father, Juzoas Narakas, was a former commander in the Air Force serving as a Deputy, and later as Home Office Minister in Lithuania. With such a family history, one would have thought young Algimantas’s destiny was sealed.

However, all went wrong one day in 1937 on a dormant WWI battlefield when, as an 11 year old boy, Nakaras was playing with a hand-grenade that exploded, severely wounding both of his lower limbs [1]. Algimantas, bleeding, but standing erect, and fearing a severe punishment, hid his wounds from his nursemaid. He could not have foreseen that his doing so would make him live in exile for a substantial portion of his young life. Chronic osteomyelitis of the left tibia and the right hip subsequently developed, and Nakaras was sent to undergo heliotherapy in the Swiss Alps (Figure 1). However, neither the sun, nor the altitude, nor the quietness of the sanatorium could heal the boy. His stay lasted for ten long years: he was unable to use his legs properly, but he was safely away from the tumult of WWII. Thus, his time was spent concentrating on developing intellectual reflexion and handskills. His flesh had yielded to his disease, but his mind and spirit thrived, thanks to his imagination. His daily routine changed abruptly when Fleming discovered the first antibiotic [2]. Within a few days time, 10,000 units of penicillin had cured him of his disease. It was in 1947, at the beginning of the Cold War, that he left the sanatorium, with a new destiny ahead of him. He remembered what a nurse had told him one day: “*Your country has vanished from all maps, nobody knows what has become of your parents, you will forever be a cripple, but despite your myopy condition, you have a very good vision and you are very skilful: why not become a clockmaker?*” [2].

**Figure 1:**
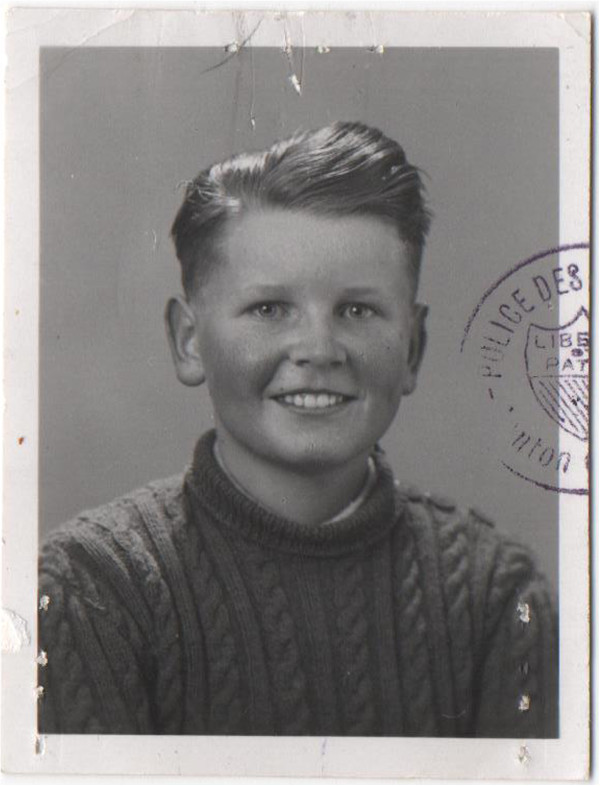
**ID picture of Narakas in the 1930’s.** Note the Swiss visa stamp. With courtesy of Diane Narakas.

Narakas began his studies at the Catholic College of Einsiedeln in Alemanic, Switzerland. There, while dissecting small animals during his science class, he became aware of his unusually sharp dexterity [3]. His health issues, and his life of privation at such a young age, gave the boy an empathy for humankind, ultimately shaping his goal to one day heal and cure people as a physician. In 1949, Narakas began his medical studies after passing his final secondary school examinations.

Narakas graduated from the Lausanne’s University Medical School in 1957. His future at last seemed assured. However, as they had many years earlier, politics once more cruelly interfered with his destiny. After German and Russian occupation, Lithuania was ceded to the Soviet block in 1944. Although unaware until he received his diploma, these political changes would ultimately deny him the right to practice medicine. Narakas became a man without a country: no longer Lithuanian, and with no possibility of becoming a Soviet (Figure 2). He would eventually wait 18 years to receive a Swiss passport. Between 1944 and 1962, his statelessness forced him to stay on in the Swiss territory, and kept him from practicing medicine.

**Figure 2:**
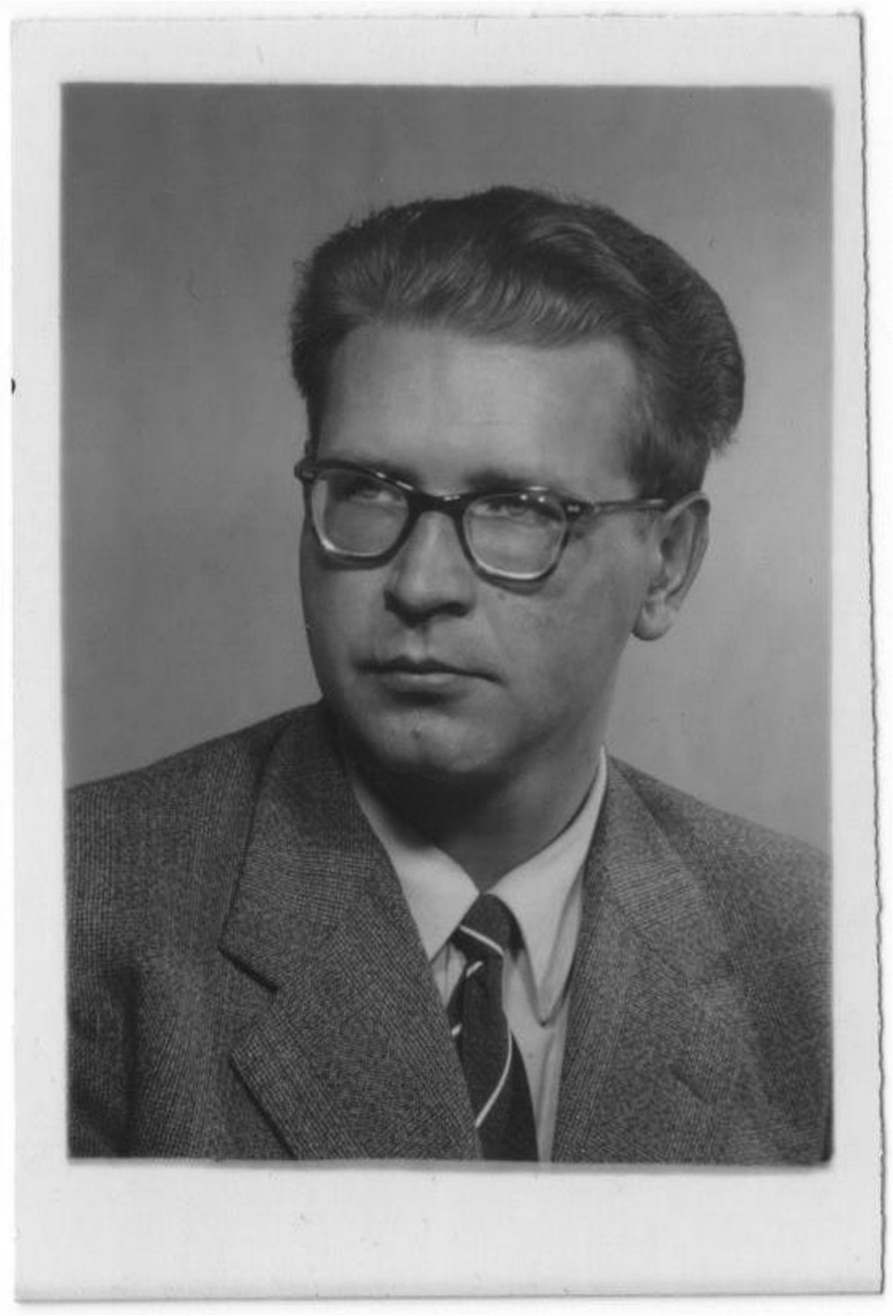
**ID picture of Narakas in the 1950’s.** Narakas was at this time an apatrid. With courtesy of Diane Narakas.

During this time, he did all his resident courses as a foreigner in the University Hospital of Lausanne [1]. He successively completed rotations in General Surgery, Neurosurgery, ENT Surgery, Cardio-Vascular and Thoracic Surgery and even in Dermatology and Internal Medicine. He also accepted other menial jobs to supplement his income: milkman, mechanic, gardener [3].

After having obtained a Swiss equivalent of his diplomas, Narakas was appointed Senior Resident and, ultimately, Consultant in Pr Verdan’s Plastic, Reconstructive, Hand and Peripheral Nerve Surgery Department. In Verdan’s Department, he was initiated to peripheral nerve microsurgical repair [4], the latter technique having been recently introduced in Europe by Michon [5]. It was the Golden Age of microsurgery, with its advancements and difficulties. Narakas never dismissed the fact that his first hand replantation failed after only one week. At 42 years old in 1969, he was appointed Specialist in General Surgery. Recognition by his peers was accorded to him in 1978 when he was bestowed the Chair of Assecurology, a discipline remote from surgery. He rejoined Pr Verdan at the Clinique Longeraie, before becoming the director of the clinic in 1981. It was only in 1989 that Hand Surgery became recognized as a separate discipline in Switzerland [1]. At 62, four years before his death, Narakas at last became a hand surgeon.

In the first half of the twentieth century, the treatment of brachial plexus injuries was limited to tendinous transfers or elbow disarticulation with shoulder arthrodesis [6,7]. All surgical attempts at nerve repair were total failures [8-11].

The interfascicular nerve graft principles described by Millesi in 1966 dramatically improved the prognosis of brachial plexus lesions. Millesi theorized that utilizing a graft to span a nerve gap would provide two tensionless junctions and would be a lesser obstacle to axonal regrowth than a primary anastomosis under tension [12]. Again, it was Millesi who achieved the first microsurgical graft repair of a brachial plexus [12-14]. Many surgical teams followed: Alnot [15] and Allieu [16] in France, Ikuta and Tsuge in Japan [17] and of course Narakas in Switzerland. Narakas reported his first case of brachial plexus repair in an emergency case in October 1969. At that time, he addressed vascular and nerve lesions in a patient presenting with acute ischaemia of the upper limb and infraclavicular brachial plexus lesions. He performed a nerve graft using the ulnar nerve. The procedure lasted 13 hours [2]. Narakas’ results confirmed Millesi’s work on nerve repair by interfascicular growth as his clinical outcomes were found satisfying even when dealing with extensive soft tissue loss [18,19].

Allieu and Narakas first met in 1972 during the XIIIth National Spanish Congress under the presidency of Santos Pallazi. Allieu, a French pioneer in brachial plexus surgery, was at first mistaken for “the assistant of Pr Allieu” by Narakas because of his young age (Figure 3). During that first meeting, they spent the entire night discussing the possibility of performing neurotization and interfascicular nerve grafts in brachial plexus surgery. Hence began their regular and fruitful collaboration.

**Figure 3:**
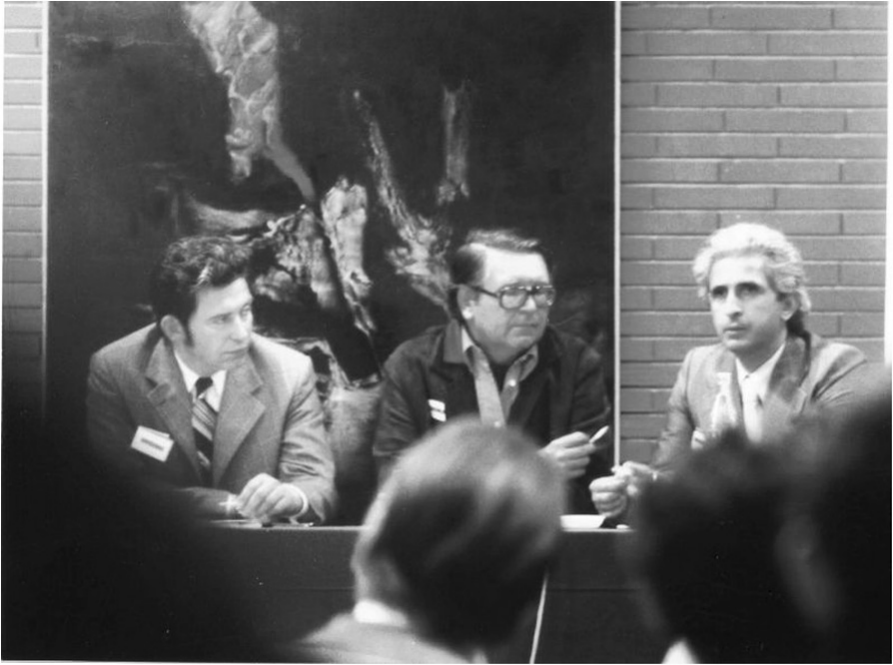
**Congress on Brachial plexus organized by Narakas in Lausanne in the early 70.** One of his first meetings with Allieu took place on this occasion. With courtesy of Yves Allieu.

Along with Alnot, Narakas was convinced that early surgery was mandatory to allow optimal nerve repair. Alnot and Narakas advocated prompt brachial plexus repair, opposing almost all leading surgical experts at the moment. During this period, optimal timing for microsurgical repair was thought to be between one and three months post-injury [20,21].

Narakas was also one of the first, with Gilbert, to use biological glue [22]. However, unlike Gilbert, Narakas systematically added two stitches to the anastomosis to enhance both nerve orientation and mechanical resistance of the suture. The duration of surgical intervention was dramatically decreased, in large part thanks to the biological glue, while increasing the overall success of the microsurgical procedure [23].

Initiated by Seddon, neurotization procedures were widely promoted by Narakas when dealing with brachial plexus avulsions. Narakas particularly favored intraplexualneurotization to address lesions involving root avulsions. Indeed, he believed functional outcome would prove better as intraplexualneurotizations involved a greater amount of motorneurons [19,24].

Narakas was the first to introduce a minimally invasive surgical concept for brachial plexus surgery. He studied various endoscopic surgical approaches in animals [25]. Only several decades later would his work be applied to human practice [26,27].

Towards the end of his career, Narakas reviewed and categorized his unique clinical experience and published the results of the surgical treatment of greater than one thousand brachial plexus injuries [4,28]. He thus developed the epidemiological rule on intraplexal lesion causes, the “7 times 70%” rule: 70% of cases involved road and street accidents, 70% of these accidents, motorcyclists were the victims, 70% of these victims presented with combined injuries, 70% of these lesions were supraclavicular, 70% of these lesions involved at least one nerve root avulsion, 70% of these avulsions involved a C7-C8-T1 level nerve root, and 70% of these avulsions developed chronic pain.

Narakas’ work comprised over 200 publications dealing with upper limb surgery, ranging from brachial plexus to epicondylitis to gleno-humeral dislocation. He collaborated on numerous scientific journals, and was an honorary member of 25 international and national medical and surgical societies. Narakas held an annual gathering of international specialists dealing with brachial plexus surgery. After his death, the meeting was named the “Narakas Club” and is held every three years.

Algimantas Otanas Narakas spent much of his young life living in exile, partly due to his severe boyhood injuries and partly due to the loss of his aristocratic status and traditional homeland. Yet, thanks to his outstanding resilience, he helped introduce the discipline of brachial plexus surgery.« If I were not desperate to do better, how would I know what hope is? » *AON*

## Competing interest

The authors declare that they have no competing interest.

## Authors’ contribution

CT: collection data. AT: collection pictures. PL: conception of the manuscript. EN: translation of the manuscript from French to English. All authors read and approved the final manuscript.
